# Synthesis of phosphorus, sulfur and silicon-containing flame retardant via thiol-ene click reaction and its use for durable finishing of cotton fabric

**DOI:** 10.1038/s41598-024-71071-5

**Published:** 2024-08-29

**Authors:** Anna Szymańska, Marcin Przybylak, Michał Dutkiewicz, Hieronim Maciejewski

**Affiliations:** 1 Poznań Science and Technology Park, Adam Mickiewicz University Foundation, Rubież 46, 61-612 Poznań, Poland; 2 Faculty of Chemistry, Adam Mickiewicz University, Uniwersytetu Poznańskiego 8, 61-614 Poznań, Poland

**Keywords:** Chemistry, Materials science

## Abstract

The article presents a very simple method of synthesis and application of a halogen and formaldehyde free, P, S and Si-containing flame retardant for the durable finishing of cotton fabric. The compound was obtained as a result of the thiol-ene click reaction. The compound was designed to have two functional groups: an alkoxysilyl group for cross-linking and binding to the cotton fabric, and a phosphate group to provide flame retardant properties. The flame retardant was applied to cotton fabric using the sol–gel method. According to the pyrolysis-combustion flow calorimetry (PCFC) technique, the use of the obtained silane for cotton treatment can reduce heat release rate (HRR) to almost 75% compared to the raw fabric. FT-IR analyses and SEM images indicate that impregnated cotton samples were covered with a layer of silanes. The SEM–EDS analysis confirmed successful modification of cotton fabrics. After 10 cycles of washing it was found that the created coatings are resistant to washing and retain their flame retardant properties.

## Introduction

The cotton fabric is characterized by excellent properties, such as comfort, softness, warmth, eco-friendliness, biodegradation, biocompatibility, dyeability, moisture absorption, breathability^1–4^. On the other hand, cotton fabric ignites very quickly and is highly flammable. Pure cotton fabric is characterized by very low the limiting oxygen index (LOI) value (about 18%) and low combustion temperature/self-ignition temperature (STI) (360–425 °C). Pure cotton also does not exhibit self-extinguishing behavior^5^. Due to the increasing restrictions regarding fire safety laws about textiles, this drastically limits the possibility of its use. At the same time, cotton fabrics are widely used in the production of clothing and decorative and technical fabrics, such as upholstered furniture, curtains, mattresses and bedding. Therefore, it is important to study novel flame-retardant agents in order to reduce fire hazards^6,7^. The flame retardant treatment of fabrics is necessary to broaden their applications, but above all to improve consumer safety^8^. In addition to the flame retardant aspect, another important factor requiring development is to achieve high durability to washing^9^. Currently, halogen free and formaldehyde free solutions are developed for flame retardant systems for cotton fabrics. Moreover, ideally the synthesis procedure should not be complicated and the modification process should not be complex and multi-stage. The aim is also to ensure that the coatings are able to strongly bond to the fiber surface and maintain crucial properties, such as comfort, aesthetics and breathability^6^.

There are several known methods of fabric modification, including chemical grafting^10–12^, layer-by-layer assembly^13,14^, and the sol–gel process^15,16^. Among them, the sol–gel method has many advantages, including the ability to create a permanent bond with the substrate, the production of only alcohol and water as by-products, and the possibility of producing multifunctional coatings^17^. Organofunctional silane coupling agents (SCA) are readily used to modify the surface of cotton fabrics due to the possibility of bonding with the hydroxyl groups of cellulose. As a result of hydrolysis and condensation, formation of the covalent bonds with the cellulosic fibers, Si–O–cellulose and flame retarded Si–O–Si network structures are formed^18^.

Silicon-containing flame retardants (FR) are popular due to several advantages they offer. One of them is its non-toxic nature, meaning that they are harmless to humans and the ecosystem. As a result of the combustion of silicon compounds, SiO_2_ is formed, which is characterized by high thermal stability (melting point 1600–1700 °C), which in turn can protect the polymer matrix against further degradation at higher temperatures. During combustion a silicaceous, compact and dense char layer covering the cotton fabric surface is formed. Such an inorganic barrier may exhibit self-extinguishing behavior^1,19,20^. On the other hand, modern phosphorus chemistry plays a crucial role in halogen free flame retardancy. Phosphorus-based flame retardants (P-FRs) act in both gas and condensed phases and are highly effective at low loadings^21^. P-FRs are characterized by multiple fire retardant mechanisms, described in many papers^22,26^. P-FRs are commonly used for their exceptional flame retardant properties. However, integrating multiple elements into a single FR system is becoming increasingly popular to enhance the performance of components like phosphorus through synergistic effects^22,23^.

Various flame retardants containing phosphorus and other elements used in the sol–gel process have been developed. Liu et al.^1^ have developed silicon, phosphorus and nitrogen containing di-(trimethoxysilylpropyl) spirocyclic pentaerythritol bisphosphorate. The compound was obtained in two steps using phosphorus oxychloride as a source of phosphor. Liu et al. have also obtained multifunctional fire retardant and hydrophobic modified cotton fabrics using poly(tetramethylcyclosiloxyl spirocyclic pentaerythritol diphosphonate) (PCTSi)^24^ or liner piperazine/phosphorous/polysiloxane copolymer^25^. Zhou et al.^26^ obtained Si, P and N containing flame retardant using (3-aminopropyl)triethoxysilane and diethyl chlorophosphite. This group also confirmed covalent binding of alkoxysilane to the cellulose fabric by XPS analysis. Castellano et al.^27^ synthesized a Si, P and N containing coating to enhance the flame retardancy of cotton fabric. In their research, the compound was obtained using (3-glycidyloxypropyl)triethoxysilane and *N*-(phosphonomethyl)iminodiacetic acid (PGPTES). Tetraethoxysilane (TEOS) was used as silane linker. The good flame retardant action of DOPO (9,10-dihydro-9-oxa-10-phosphaphenanthrene-10-oxide) derivatives has also been confirmed^28^. Vasiljević et al. synthesized DOPO-modified vinyl trialkoxysilane^29^. Zhang et al.^30^ synthesized a compound containing piperazine link between alkoxysilane group and DOPO, named 1-(9,10-dihydro-9-oxa-10- phosphaphenanthrene-10-oxide)-4-(trimethoxysilylmethyl) piperazine (DOPO-PiP-Si). Polyphosphazenes-based compounds are another group of derivatives providing high efficiency flame retardant effect^31^. Dutkiewicz et al. modified cotton fabric with phosphazene containing 6 alkoxysilyl groups, obtained by hydrosilylation of cyclic phosphazene substituted with allyloxy groups^9^. Triazine-based compounds also deserve attention as FRs^32^.

Thiol-ene click reaction is often used for the synthesis of FR materials^33–35^ and can also be used for attaching flame retardant on the surface of fabrics by reaction between SH-group containing cotton and alkenyl groups of the flame retardant^36^. The effect of the thiol-ene reaction is the introduction of a sulfur atom into the structure of the compound, which may contribute to a beneficial synergistic effect with the remaining atoms.

The aim of this study was a synthesis of a flame retardant compound and its use for modification of cotton fabric. In the present work silane containing phosphorus, sulfur and silicon atoms was obtained as a result of the thiol-ene click reaction. The obtained compound was used to modify cotton fabric through the sol–gel process to create a durable siloxane coating on the cotton surface.

## Methods

### Materials

*O*,*O*′-diethyl dithiophosphate (technical grade, 90%) was purchased from Sigma-Aldrich. Triethoxyvinylsilane (97%) and 2,2-dimethoxy-2-phenylacetophenone (DMPA, 99%) were purchased from Aldrich. Chloroform-d (99.8% + Ag (CDCl_3_)) was purchased from Deutero. Sodium hydroxide, tetrahydrofuran and acetic acid were purchased from Chempur. A bleached cotton fabric with an areal density of 145 g/m^2^ was supplied by the Textile Factory in Łódź (Poland).

### Synthesis of flame retardant: *O,O'*-diethyl-*S*-[(2-triethoxysilyl)ethyl] phosphorodithioate

**Fig. 1:**
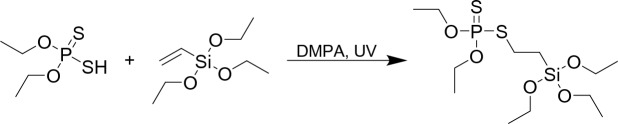
Synthesis of flame retardant via hydrothiolation reaction.

^**1**^**H NMR** (400 MHz, CDCl_3_) δ(ppm): 0.97–1.07 (m, 2H, SiCH_2_); 1.16 (t, *J* = 7.1 Hz, 9H, Si(OCH_2_CH_3_)_3_; 1.29 (t, *J* = 6H, P(OCH_2_CH_3_)_2_; 2.84–2.97 (m, 2H, SCH_2_); 3.76 (q, *J* = 7.0 Hz, 6H, Si(OCH_2_CH_3_)_3_; 3.99–4.20 (m, 4H, P(OCH_2_CH_3_)_2_. ^**13**^**C NMR** (101 MHz, CDCl_3_) δ(ppm): 13.40 (d, *J* = 5.1 Hz, SiCH_2_); 15.82 (d, *J* = 8.4 Hz, P(OCH_2_CH_3_)_2_; 18.23 (Si(OCH_2_CH_3_)_3_; 28.80 (d, *J* = 4.2 Hz, SCH_2_); 58.54 (Si(OCH_2_CH_3_)_3_; 63.64 (d, *J* = 5.9 Hz, P(OCH_2_CH_3_)_2_. ^**29**^**Si NMR** (79 MHz, CDCl_3_) δ(ppm): − 49.92 (d, *J* = 1.7 Hz). ^**31**^**P NMR** (162 MHz, CDCl_3_) δ(ppm): 94.65.

A round-bottom flask equipped with a magnetic stirrer was loaded with *O*,*O*′-diethyl dithiophosphate 2.593 g, (12.531 mmol, 90% purity), triethoxyvinylsilane 2.709 g (14.092 mmol, 99% purity) and 2,2-dimethoxy-2-phenylacetophenone 0.032 g (0.125 mmol). The mixture was stirred until the photoinitiator dissolved and was then irradiated with a UV lamp for 2 h. The excess of silane was removed under reduced pressure. The product was purified by trap-to-trap distillation to 4.227 g and yield 89.6% (Fig. 1).

### Modification of cotton fabrics

The mercerization process involves immersing cotton fabrics in a 10% aqueous solution of sodium hydroxide for 10 min at room temperature. After the treatment, the fabrics are thoroughly rinsed in water to remove any residual sodium hydroxide.

The modification process of cotton fabrics involved preparing three silane solutions with concentrations of 2.5%, 5%, 10% and 20%. The appropriate amount of silane was mixed in a flask, with 5% by weight of acetic acid, 5% by weight of water, and tetrahydrofuran as the solvent. A one-hour hydrolysis process was then conducted. Following hydrolysis, the solution was transferred to trays, where both mercerized and non-mercerized fabrics were impregnated for one hour. After impregnation, the fabrics were removed, excess solvent was squeezed out, and the fabrics were dried at a temperature of 80 °C. Finally, the fabrics were cured for 3 min at a temperature of 130 °C. The cotton samples were weighed before and after modification and add-on percent was calculated using Eq. (1).

1 $$Add-on \left[\%\right]= \frac{{mass}_{after treatment}-{mass}_{before treatment}}{{mass}_{before treatment}} \times 100$$

### Washing process

The washing process of modified fabric samples was carried out according to the standard PN-EN ISO 105-C06:2010 Textiles—Tests for color fastness—Color fastness to domestic and commercial laundering. The process was performed at 40 °C for 60 min. using a detergent and this was followed by rinsing.

### Analytical methods

Nuclear magnetic resonance spectra ^1^H, ^13^C and ^29^Si NMR were recorded at 298 K on Bruker Avance III HD 400 MHz spectrometers. CDCl_3_ was used as a solvent. Fourier transform infrared (FT-IR) spectra of the polymers were taken on a Bruker spectrometer, model Tensor 27, equipped with a SPECAC Golden Gate diamond ATR (Attenuated total reflection) unit. In each case, 16 scans were collected for each spectrum at the resolution of 2 cm^−1^. Photoinitiated thiol-ene click reaction was performed using a 150 W medium pressure mercury lamp, emitting a 280–600 nm wavelength (LQ400 mercury lamp, Gröbel UV-Elektronik GmbH). The flammability was assessed using a pyrolysis-combustion flow calorimetry (PCFC) technique. All analyses were performed on Fire Testing Technology Ltd, FTT0001 MICROCAL. The heating rate (β) was 1.25 °C s^−1^, the pyrolysis temperature range 75–750 °C, and the combustion temperature 900 °C. The gas flow was a mixture of O_2_/N_2_ 20/80 cm^3^ min^−1^ and the sample weight 4–5 ± 0.01 mg. The heat release temperature (T_max_), the heat release capacity (ηc), and the maximum specific heat release rate (HRR_max_) were derived from calorimetric measurements. The tests were repeated three times and the experimental error in HRR (heat release rate) was ± 2% and the instrumental error in T was 1 °C and t was 1 s. The flammability properties, in terms of the limiting oxygen index (LOI), were determined according to ISO 4589-2, with three measurements per sample. Thermogravimetric analysis (TGA) of 9–10 mg cotton fabric samples was performed using a TA Instruments Q50 TGA thermobalance at a heating rate of 10 °C/min from room temperature to 700 °C under nitrogen. A vertical burning test was conducted on 7.5 cm by 30 cm cotton samples according to ASTM D6413. The elemental composition of the applied coating, specifically C, O, S, Si, P, was determined using SEM–EDS with a Hitachi SU-3500 electron scanning microscope (SEM) with an EDS attachment (Poznań, Poland). The bending length (stiffness) of cotton fabric samples was measured followed the ASTM D 1388. Samples were cut to the size of 2.5 cm × 20 cm. Samples were placed on a horizontal platform and slid until their leading edges projected from the edge to form a 41.5° angle with the horizontal. The overhang length (*O*) was recorded. Tests were done with 5 samples tested for each fabric type. Bending length (c) and flexural rigidity (G) of samples were determined according to Eqs. (2) and (3).

2 $$ c \, = O/2 \, \left[ {{\text{mm}}} \right] $$

3 $$ G \, = \, 1.421 \times {10^{ - 5}}\cdot W \cdot{c^3}\left[ {{\mu J}/{\text{m}}} \right] $$

C is the bending length [mm], W is the fabric unit mass [g/m^2^].

## Results and discussion

**Fig. 2:**
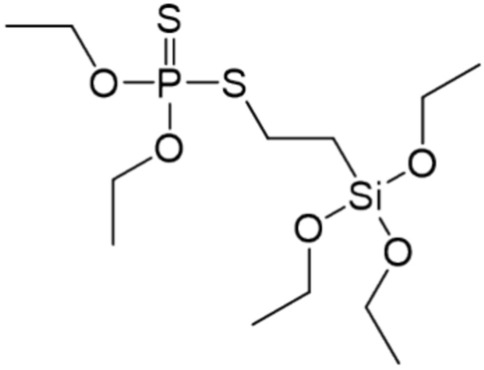
Structure of flame retardant.

During this study, a P, S and Si-containing flame retardant was used for modification of cotton fabric (Fig. 2).

**Fig. 3:**
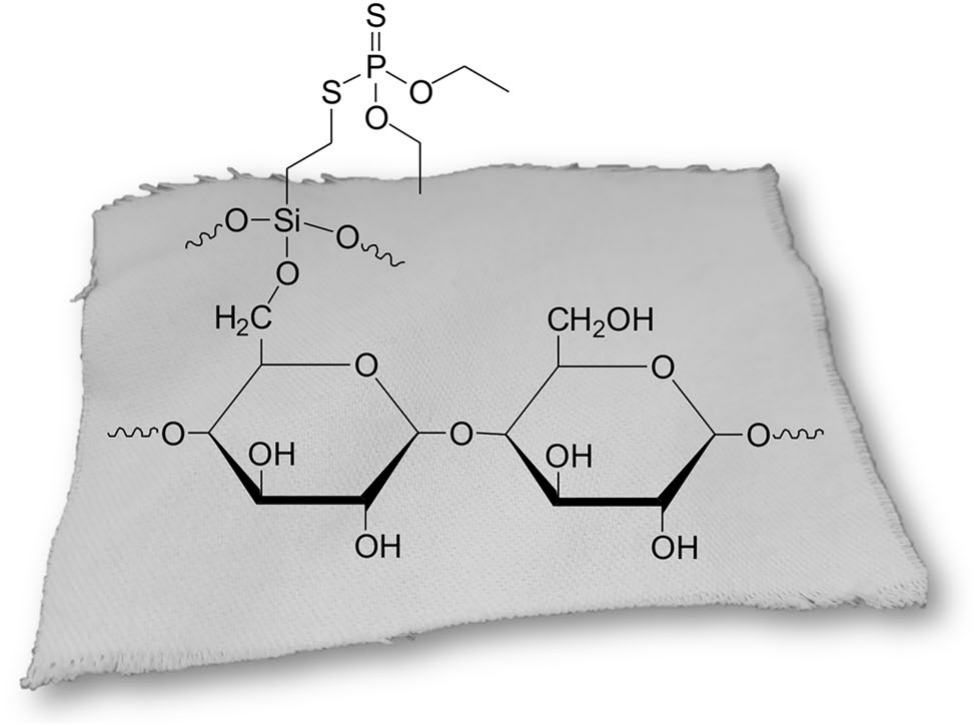
Proposed structure of modified cotton fabric (cellulose) by silane via sol–gel process.

The compound was obtained as a result of the thiol-ene click reaction via the addition of SH group-containing phosphate to a HC=CH_2_ bond of triethoxyvinylsilane in the presence of a photoinitiator and UV irradiation. The method of synthesis was simple and efficient, and the product obtained with almost 90% isolation yield (NMR and FT-IR spectra are included in Supplementary Information). The obtained derivative was used for modification of cotton fabric via sol–gel process. During this process triethoxysilyl groups of synthesized silane were firstly hydrolyzed by water in acidic condition to silanol groups and next condensed to form a crosslinked network on the cotton fabric surface. A schematic illustration of modified fabric is shown in Fig. 3.

During the study mercerized and non-mercerized cotton fabrics were treated. Table 1 shows the method of sample modification and their add-on values.

**Table 1:** Explanation of sample symbols and add-on values.

Sample	Mercerization	Silane concentration (%)	Add-on value (%)
S2.5	−	2.5	4.1
S2.5M	+	2.5	4.4
S5	−	5	6.2
S5M	+	5	7.1
S10	−	10	12.1
S10M	+	10	13.4
S20	−	20	19.4
S20M	+	20	21.4

Analyzing the data presented in Table 1, it can be seen that all modified fabrics have relatively low add-on values. The add-on value increases with the silane concentration in the modifying compositions. Mercerized fabrics are characterized by slightly higher add-on values compared to analogous non-mercerized samples. The mercerization process activates the surface of cotton fibers and creates –O–Na + active centers, which promotes the formation of bonds between the modifier and cellulose. Relatively low add-on values are very advantageous from both an economic and functional point of view.

**Fig. 4:**
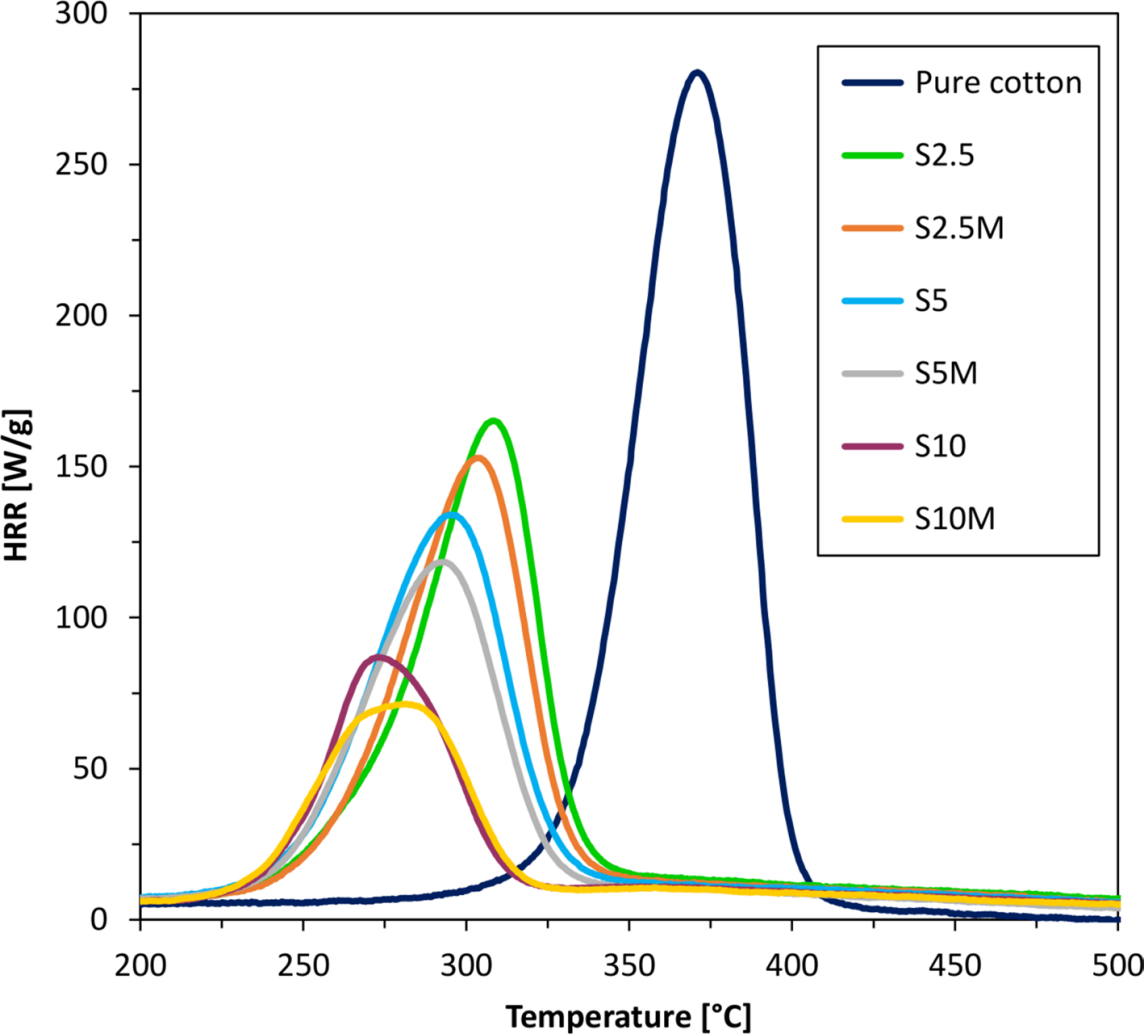
Heat release rate (HRR) of samples before washing process.

The cotton samples (2.5, 5 and 10 wt.% silane concentration) were analyzed with microcalorimeter PCFC. Heat release rate (HRR) curves of samples before washing process are presented in Fig. 4.

**Fig. 5:**
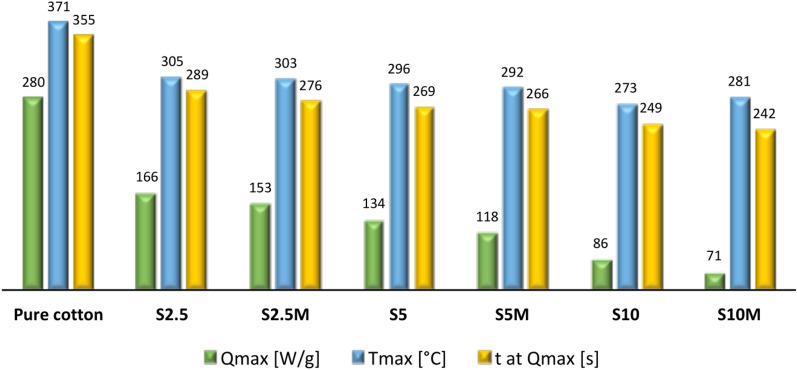
PCFC analyses of modified samples before washing process.

Figure 5 shows the remaining data obtained during the microcalorimetric analysis, including: the maximum rate of heat release (Q_max_), the temperature of reaching the maximum rate of heat release (T_max_) and the time of achieving the maximum rate of heat release (t at Q_max_).

The results presented in Figs. 4 and 5 show that the applied modifications significantly reduced the HRR value compared to the unmodified fabric. Samples treated with a 2.5% silane solution are characterized by a HRR reduction of over 40% compared to the raw fabric. This is a very large difference in the heat release rate at an extremely low concentration of the modifying composition and the add-on value of the sample. The curves presented in Fig. 4 illustrate that as the concentration of the composition increased, the modifying heat release rate values decreased. Mercerized fabric impregnated with a 10% silane solution (S10M) shows a HRR reduction of almost 75% compared to the raw fabric. Moreover, the obtained results showed that mercerized samples after the impregnation process had slightly lower heat release rate values compared to analogous, non-mercerized samples. The mercerization process activated the hydroxyl groups of cellulose and created –O–Na + active centers, which promoted the formation of bonds between the modifier and the fiber surface. This process had a positive effect on reducing the HRR of modified fabrics.

The findings depicted in Figs. 4 and 5 unequivocally demonstrate a notable decrease in both the temperature of maximum heat release rate (T_max_) and the time required to attain it (t at Q_max_) for the modified samples in comparison to raw cotton. This reduction represents a significant alteration in the thermal behavior of the samples. It is noteworthy that the temperature at which the maximum release rate occurs aligns with the temperature of maximum pyrolysis rate. It is crucial to emphasize the disparity between the conditions of a microcalorimeter and a cone calorimeter. Unlike the latter, where combustion of the sample takes place, a microcalorimeter operates with the sample situated in an inert gas atmosphere (N_2_). The temperature gradually escalates until reaching T_max_, at which point pyrolysis commences. Subsequently, the resultant pyrolysis products, primarily flammable gases, are introduced into the chamber where they mix with oxygen, leading to combustion. The determination of heat release rate hinges on the measurement of oxygen loss. As documented in the literature, one effective strategy to mitigate carbon dioxide emissions involves maximizing carbon residue at the expense of reducing flammable gases. It is a well-established principle that lower temperature pyrolysis fosters greater carbon residue formation, consequently diminishing the production of flammable gases. In essence, the observed reduction in T_max_ and time to reach Q_max_ signifies a mitigation of flammability, a highly advantageous outcome. Moreover, the values of T_max_ and t at Q_max_ decrease with increasing silane concentration in the modifying composition.

**Fig. 6:**
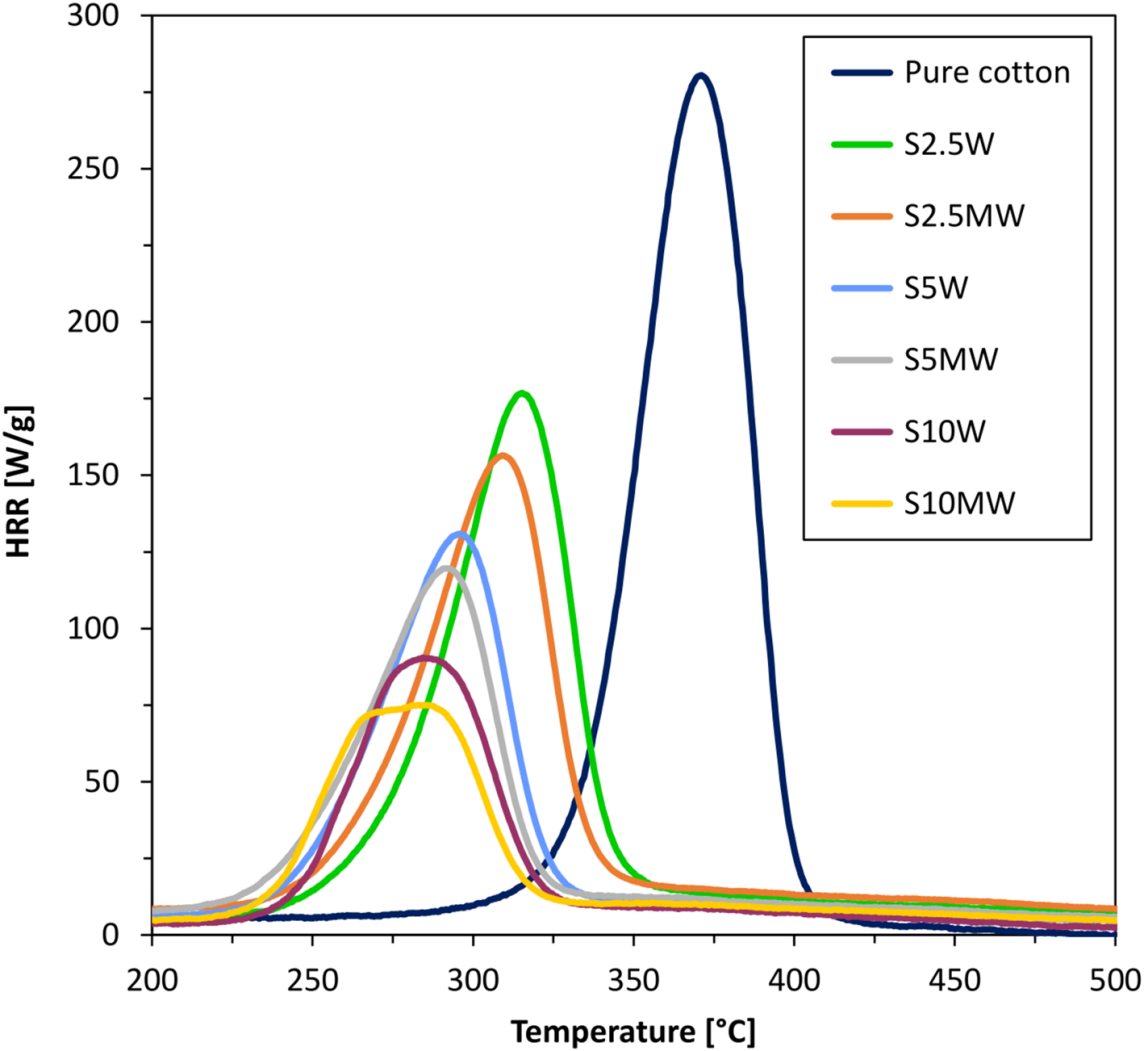
Heat release rate (HRR) of samples after 10 washing cycles (W).

**Fig. 7:**
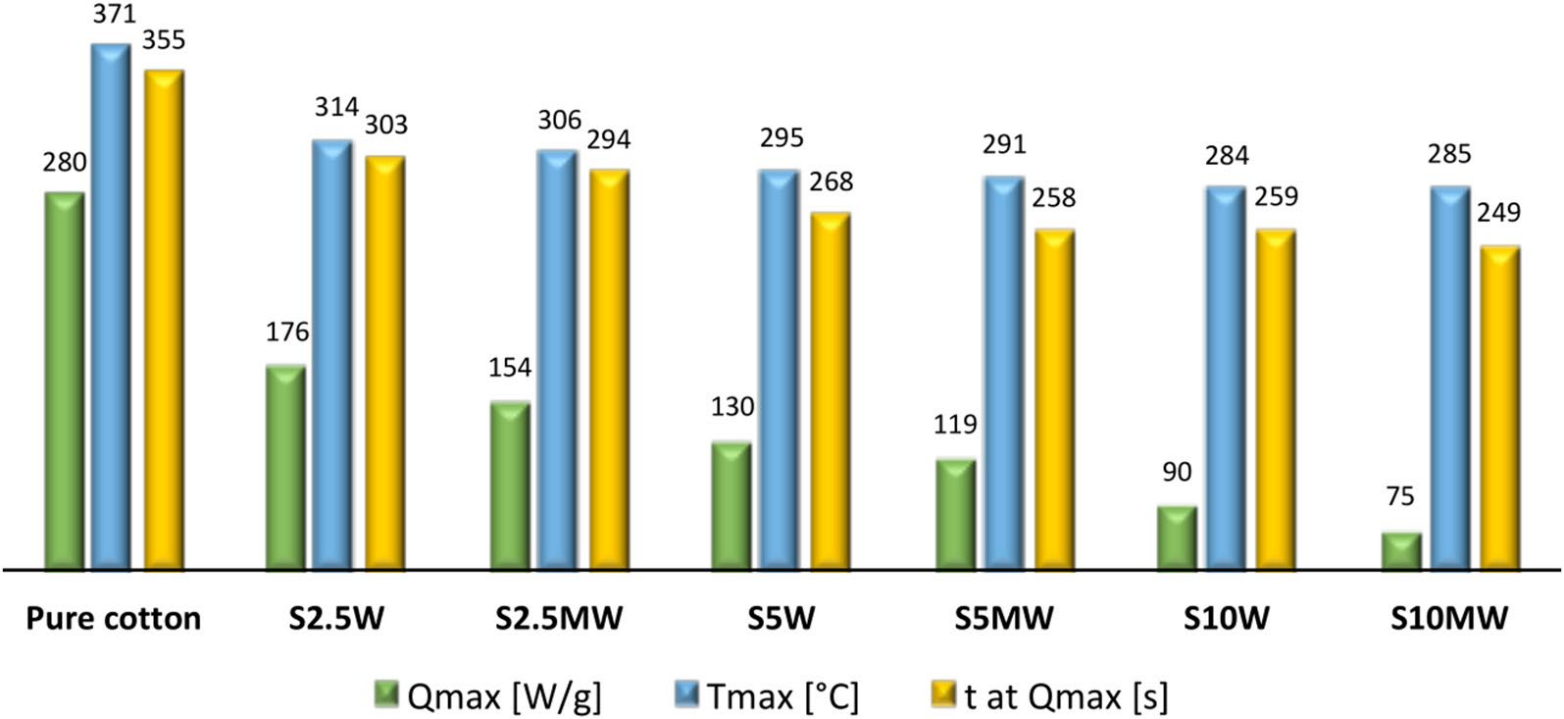
Data from PCFC analyses of samples after 10 washing cycles (W).

Samples were washed 10 times to confirm durability of modification. After 10 washing cycles, samples were analyzed on a PCFC pyrolysis microcalorimeter (Figs. 6 and 7).

One of the most challenging aspects at in fire-retardant modification of fabrics is the durability of the created coatings. A huge number of developed modifications are not resistant to washing or are only partially durable. However, due to the aspects of use, the durability of the produced coatings is extremely important. The results presented in Figs. 6 and 7 clearly confirm the durability of the developed modifications. Both the shape and course of the curves from washed fabrics are almost identical to those from unwashed samples. Putting the sample through 10 washing cycles slightly changes in the heat release rate. Also, the remaining data obtained during the PCFC analysis barely changed as a result of washing. The alkoxy groups present in the structure of the silane modifier (Si(OC_2_H_5_)_3_ were transformed into hydroxyl groups as a result of the hydrolysis process. This process enabled the formation of covalent bonds on the fiber surface. The obtained results allow us to conclude that the bonds formed are durable and the silane remains on the fiber surface even after repeated washing. The creation of durable fire-retardant coatings on the fiber surface is a spectacular success.

**Fig. 8:**
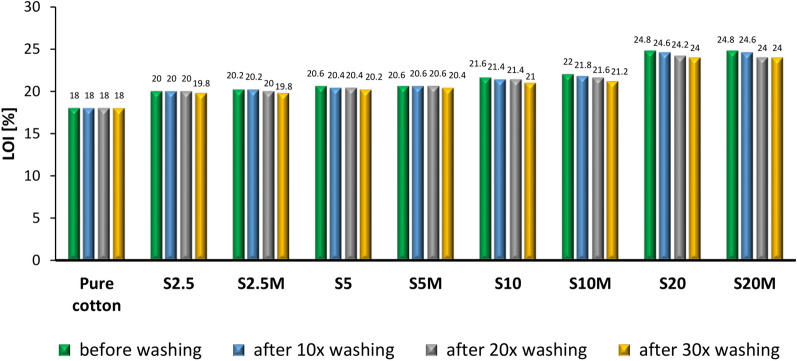
LOI of modified samples before washing and after being ten, twenty, and thirty washing cycles.

Both before washing and after undergoing ten, twenty, and thirty washing cycles, the modified fabric samples were subjected to analysis of the measurement of the limited oxygen index (LOI) (Fig. 8).

The results presented in Fig. 8 allow us to conclude that all the applied modifications increased the LOI values; however, the differences between the modified and unmodified cotton are not spectacular. The limited oxygen index values increase with the concentration of silane in the modifying composition. Figure 8 shows that mercerized samples, after impregnation with the silane solution, show slightly higher LOI values than non-mercerized. It is worth noting that although subsequent washing cycles caused a slight decrease in LOI values, the differences are not significant.

**Fig. 9:**
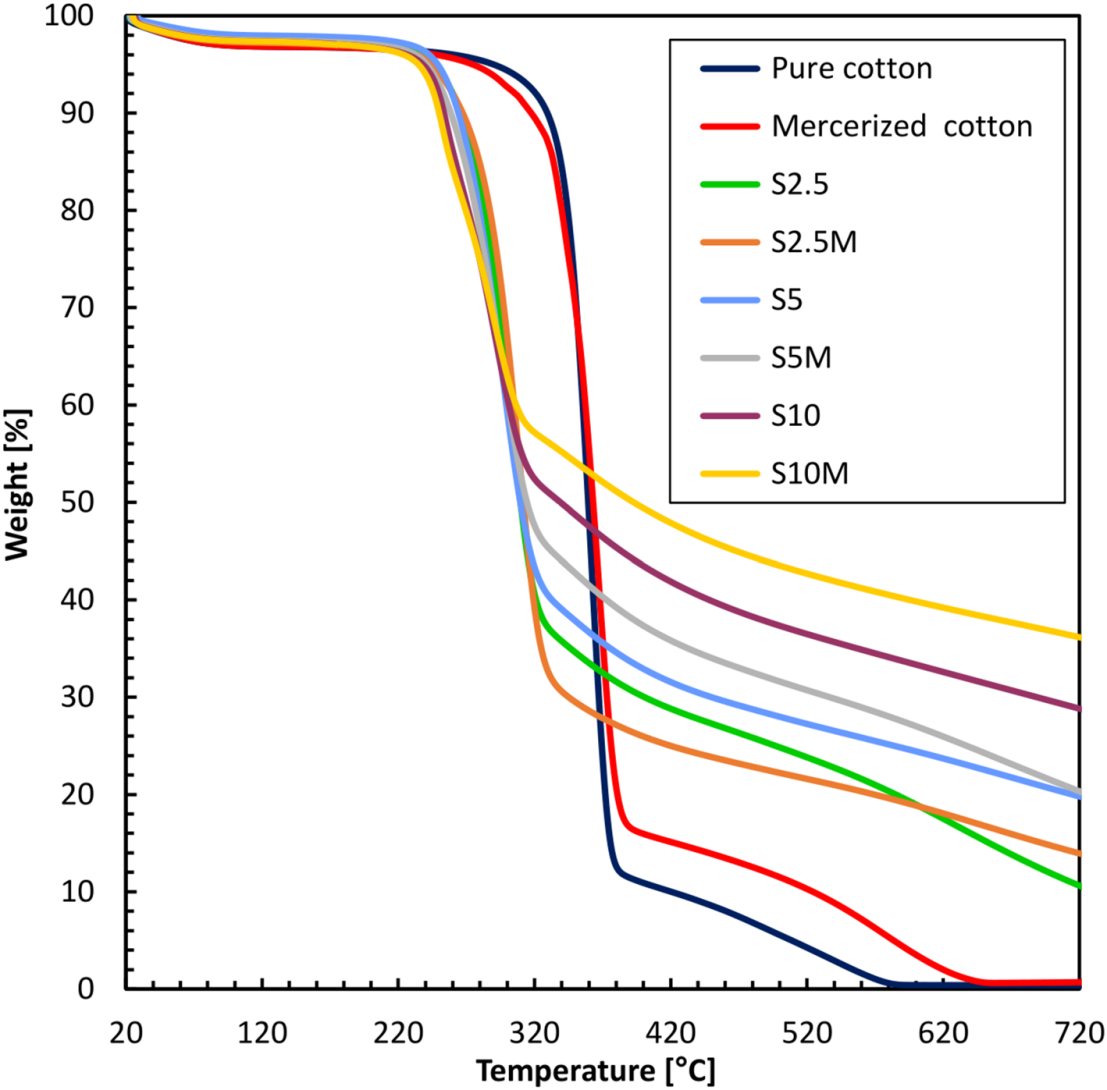
TG curves of modified samples before washing.

**Fig. 10:**
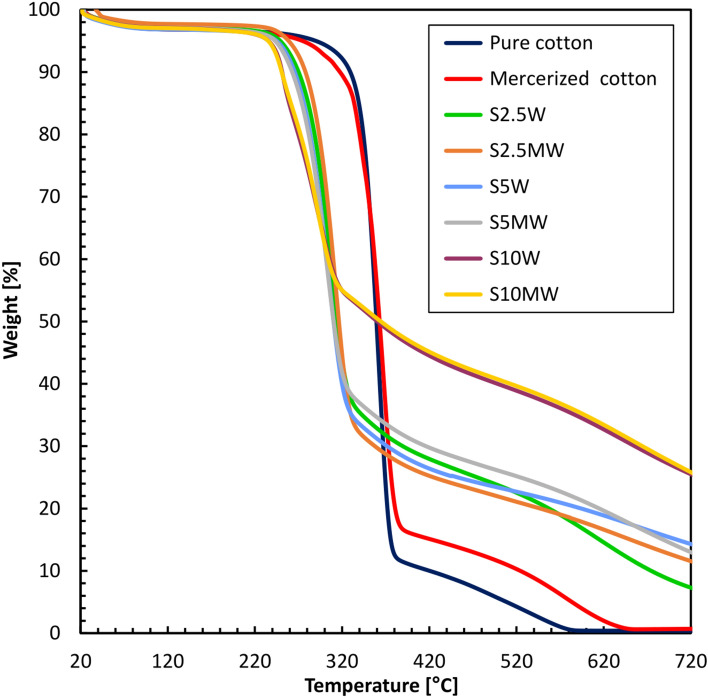
TG curves of modified samples after washing.

Next, the thermal stability of the modified and unmodified fabrics was examined. For this purpose, thermogravimetric analyses (TGA) were performed in a nitrogen atmosphere (Table 2). Thermogravimetric curves of unwashed and washed samples are shown in Figs. 9 and 10.

**Table 2:** Temperatures of mass loss of samples in the nitrogen atmosphere.

Sample	Mass loss temperature (°C)						Residue at 700 °C (%)
	5%	Δ	10%	Δ	20%	Δ	
Pure cotton	290	**–**	329	**–**	344	**–**	0.38
Mercerized cotton	275	–	317	–	340	–	0.66
S2.5	245	45	266	63	283	61	11.8
S5	248	42	264	65	279	65	20.6
S10	237	53	253	76	271	73	29.5
S2.5M	244	46	267	62	287	57	14.7
S5M	241	49	258	71	277	67	21.4
S10M	234	56	250	79	269	75	36.8
S2.5W	249	41	270	59	288	56	8.4
S5W	244	46	265	64	283	61	15.0
S10W	235	55	251	78	269	75	27.0
S2.5MW	258	32	278	51	293	51	12.5
S5MW	243	47	262	67	281	63	14.2
S10MW	235	55	250	79	269	75	36.9

The TGA analysis shows a considerably improved thermal stability for modified cotton fabrics compared to pure cotton fabrics. Pure cotton begins to degrade at 290 °C and has 0.38% residue at 700 °C. On the other hand, the modified samples present much higher values of residues, pointing to their higher thermal stability. For example, the mercerized cotton sample modified with a 10% modifier (S10M) presents the highest residue, which is 36.8%, and the washed sample (S10MW) demonstrates a residue of 36.9%. These large residue values point to the efficiency of the modification in enhancing the thermal resistance of the fabric.

As can be seen from the comparison between the washed and the unwashed samples, the thermal degradation is almost the same, which means that washing has minimal effect on the modification's efficiency. Specifically, for the S5M mass loss occurs at 241 °C, 258 °C, and 277 °C for 5%, 10%, and 20% weight loss, and a residue of 21.4%. For the S5MW washed sample, the corresponding mass loss temperatures are 243 °C, 262 °C, and 281 °C, with a residue of 14.2%; the slight differences in these values suggest that the modifier is still primarily intact after washing, thus keeping the thermal properties in the fabric. The 2.5% modified sample (S2.5) has mass loss temperatures at 245 °C, 266 °C, and 283 °C, with a residue of 11.8%, and the washed version (S2.5W) shows mass loss temperatures at 249 °C, 270 °C, and 288 °C, with a residue of 8.4%. Similarly, the 10% modified sample (S10) shows mass loss temperatures at 237 °C, 253 °C, and 271 °C, with a residue of 29.5%, while the washed version (S10W) shows temperatures at 235 °C, 251 °C, and 269 °C, with a residue of 27%. These results confirm minor changes due to washing for the 2.5% and 10% modified samples.

Mercerization also affects thermal stability, generally resulting in slightly lower mass loss temperatures but higher residues. For example, the mercerized sample with a 10% modifier (S10M) showed mass loss temperatures at 234 °C, 250 °C, and 269 °C, with a residue of 36.8%, while the non-mercerized sample (S10) showed slightly higher mass loss temperatures at 237 °C, 253 °C, and 271 °C, but a lower residue of 29.5%. This indicates that mercerization enhances the binding or incorporation of the modifier into the cotton fibers, resulting in improved thermal stability. Differences in results are minimal for the 5% modified samples, where the unwashed sample (S5) shows mass loss temperatures at 248 °C, 264 °C, and 279 °C, with a residue of 20.6%. In contrast, the washed sample (S5W) shows mass loss temperatures at 244 °C, 265 °C, and 283 °C, with a residue of 15%.

**Fig. 11:**
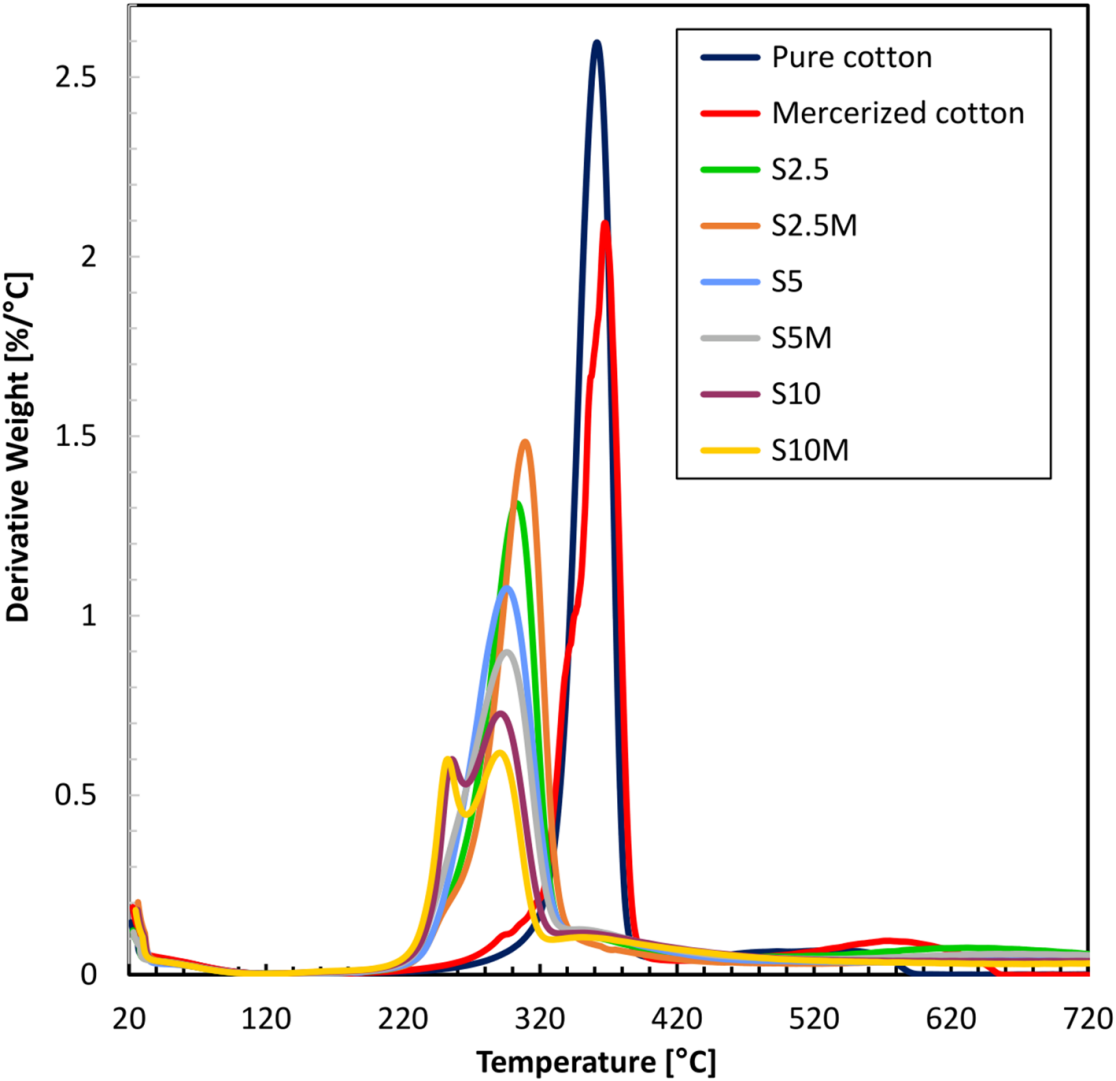
DTG curves of modified samples before washing.

**Fig. 12:**
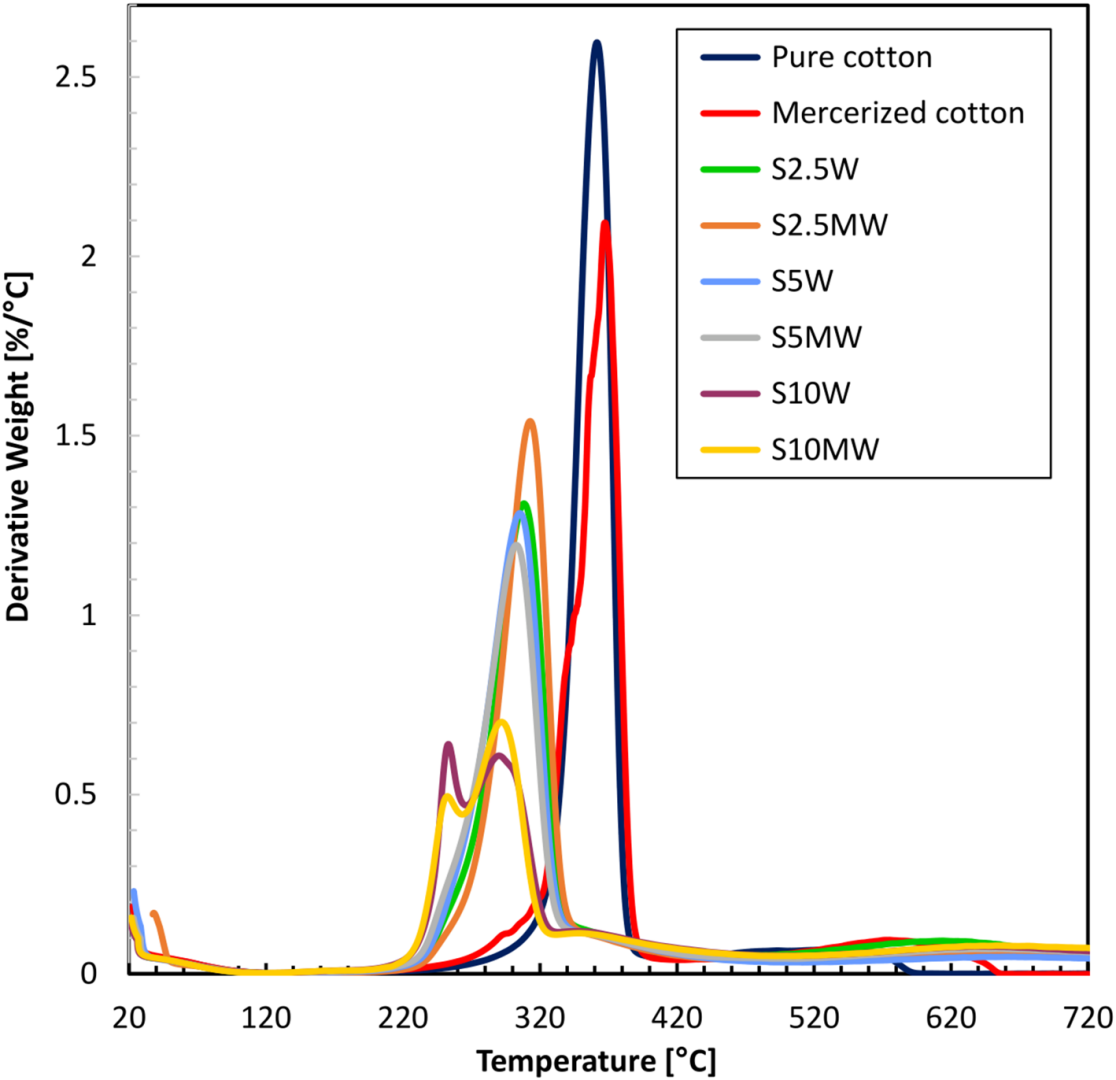
DTG curves of modified samples after washing.

Based on TG analyses, DTG derivatives were also determined. Table 3 lists the main DTG peaks and the mass loss rate they achieve. Figures 11 and 12 show the DTG curves for samples before and after washing.

**Table 3:** DTG onset and the temperatures at which it occurred in nitrogen atmosphere.

Sample	DTG main peaks			
	Temp. (°C)	(°C)	Value (%/°C)	Δ (%)
Pure cotton	361	**–**	2.6	**–**
Mercerized cotton	367	–	2.01	–
S2.5	303	58	1.31	50
S5	294	67	1.07	59
S10	293	68	0.72	72
S2.5M	308	53	1.48	43
S5M	295	66	0.89	66
S10M	252	109	0.6	77
S2.5W	308	53	1.31	50
S5W	304	57	1.28	51
S10W	289	72	0.61	77
S2.5MW	312	49	1.5	42
S5MW	302	59	1.19	54
S10MW	292	69	0.71	73

The DTG results also show significant reductions in the degradation rate of the modified fabrics compared to the pure cotton. Pure cotton has a DTG peak at 361 °C coupled with a degradation rate of 2.6%/°C. The modified samples have a much lower rate of degradation. For example, the peak of the S10M sample is at 252 °C, with a degradation rate of 0.60%/°C, and that of the washed sample (S10MW) is 292 °C, with a rate of 0.71%/°C. The degradation rate is reduced significantly with the modification treatment, enhancing thermal stability. The differences between the washed and unwashed samples are minor, a property representing modifying durability. For example, the 5% modified sample (S5M) peaks at 295 °C with a rate of 0.89%/°C; in contrast, the washed sample (S5MW) peaks at 302 °C with a rate of 1.19%/°C. The difference is, however, slight, and it may be inferred that the modifier is not heavily washed out, hence the thermal stability of the fabric is preserved. In general, the mercerized samples show lower rates of degradation compared to the non-mercerized, indicating that there is a better incorporation of the modifier. For instance, the 2.5% mercerized sample (S2.5M) has its peak at 308 °C at a rate of 1.48%/°C, while the non-mercerized sample (S2.5) has its peak at 303 °C at a rate of 1.31%/°C. In the curves derived from fabrics modified with 10% solutions, two peaks are visible. This is due to the decomposition of the modifier occurring first, followed by the decomposition of the cotton fabric. It is important to note that the decomposition of the modifier occurs at lower temperatures than the fabric, which is advantageous for flame retardants. Furthermore, these results correlate with the HRR curves.

By comparing these DTG results with TGA data, an integral concept of the thermal behavior of the fabrics is given. Considering the decreased degradation rates in the DTG, the high residues obtained by TGA at 700 °C with the modified samples are 36.8% for S10M and 36.9% for S10MW. This indicates that the existence of the modifier does not only reduce the degradation rate but also leads to higher thermal stability and a lower extent of mass loss in general. The beginning of thermal degradation in TGA lies in the data for lower temperatures of modified samples—for example, 234 °C for the S10M sample. It corresponds to DTG data whereby the modified samples, in turn, show lower maximum temperatures—for instance, 252 °C for S10M. It does improve the validity of the conclusion about the decisive impact of modification on the thermal properties of such fabrics.

TGA and DTG results show that washing had a minimum influence on thermal properties. For example, TGA degradation of 21.4% and 14.2% for S5M and S5MW, respectively, with the same degradation trend in the DTG (0.89%/°C for S5M and 1.19%/°C for S5MW), the results are proofs that the modification is stable to washing. It is possible to sum up that the results obtained from thermogravimetric and derivative thermogravimetric analyses underline the positive influences of modifying cotton fabrics. The modification led to considerably less degradation and higher residues, proving higher thermal stability. The slight differences between washed and unwashed samples further underline the durability of the modification. Lower onset temperatures are found only in mercerized samples but are thus associated with better incorporation of the modifier and therefore better thermal features. This further underlines the efficiency of the chemical method in increasing the thermal performance and durability of cotton fabrics.

**Fig. 13:**
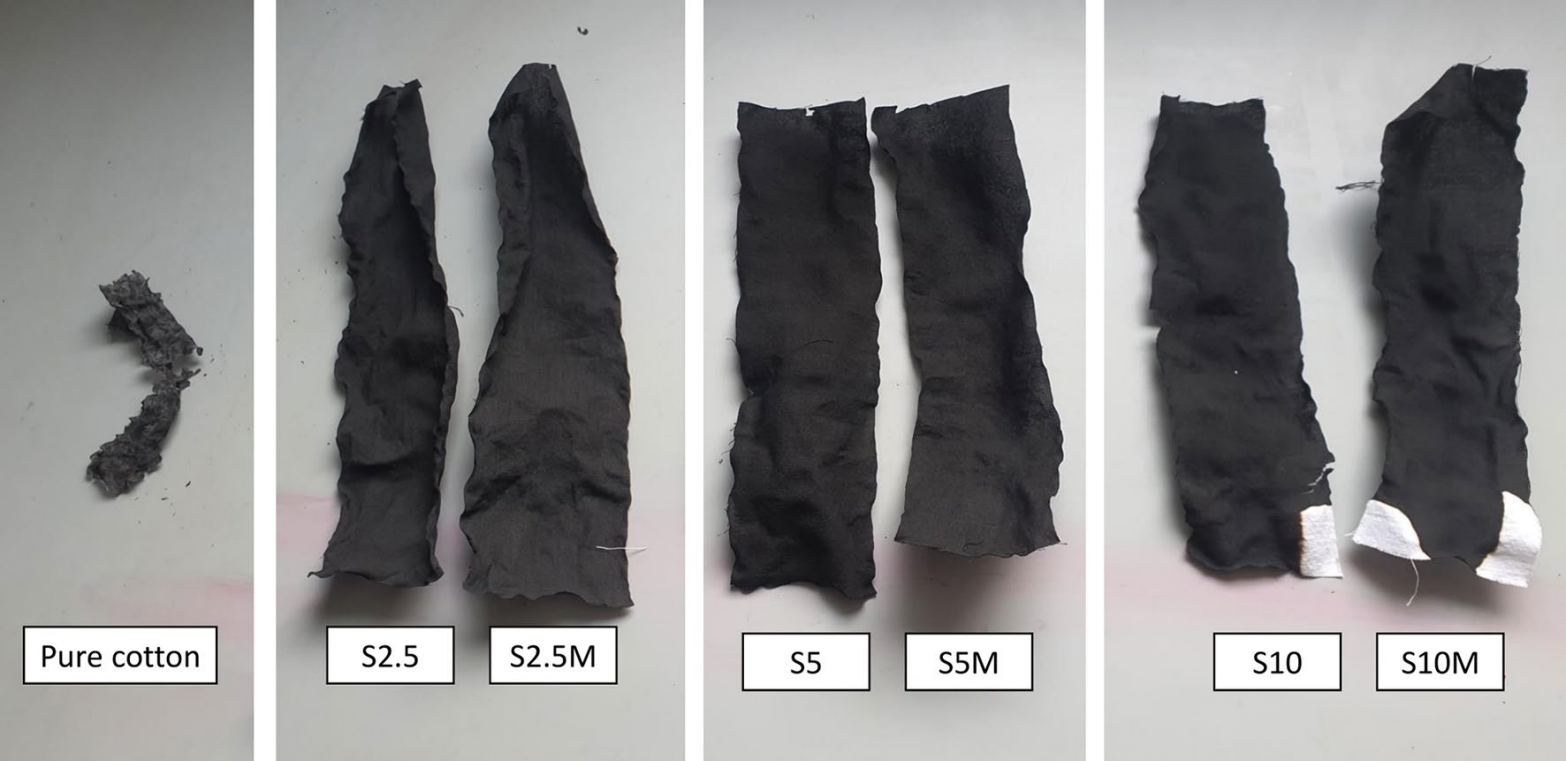
Vertical burning test results of modified samples.

Subsequently, vertical burning tests were performed on the modified samples. The test results are presented in Fig. 13 and Table 4.

**Table 4:** Vertical burning test results of modified samples.

Sample	After-flame time (s)	After-glow time (s)	Char length (mm)
Pure cotton	8	30	–
S2.5	14	0	300
S2.5M	16	0	300
S5	20	0	300
S5M	21	0	300
S10	23	0	300
S10M	23	0	300

The modifications resulted in significant changes in the burning process of the samples compared to the raw cotton fabric. It is evident that the modified fabrics exhibit a much larger char residue after the burning process, whereas the raw fabric burned completely. Moreover, for samples modified with the highest concentration of silane, unburned fragments of the fabric are visible. It is noteworthy that modified fabrics have a zero after-glow time. Additionally, the burning time was extended compared to the unmodified fabric.

**Fig. 14:**
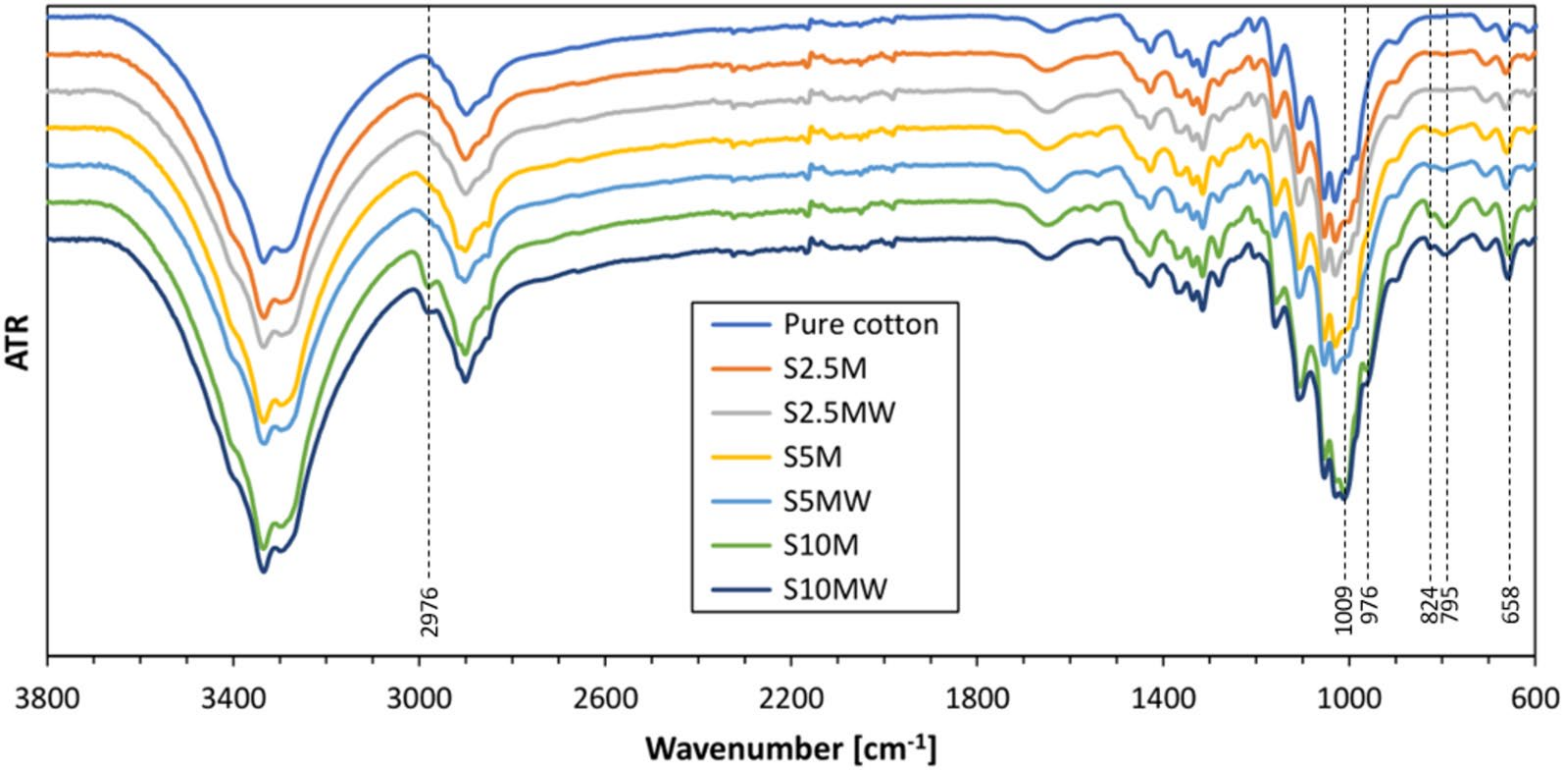
FT-IR spectra of a raw cotton fabric and modified cotton fabrics (S2.5M, S2.5MW, S5M, S5MW, S10M and S10MW).

The samples were subjected to FT-IR analyses to confirm the effectiveness of the modification (Fig. 14).

The FT-IR spectra of pure cotton fabric and silane-modified cotton fabrics (S2.5M, S5M and S10M washed and non-washed) are shown in Fig. 14. The spectra of modified samples show the bands at about 2976 cm^−1^ coming from CH stretching vibration of CH_3_. The spectra show bands coming from POCH_2_CH_3_, P=S and P–S of organophosphorus group at about 1009 cm^−1^, 976 cm^−1^, 824 cm^−1^, 795 cm^−1^ and 658 cm^−1^. These bands are the most intense for samples modified with the highest silane concentration of 010%, both unwashed and washed (S10M and S10MW) and invisible in the spectra for samples modified with a concentration of 2.5%. The bands in the range 950–1100 cm^−1^ are attributed to the characteristic peaks of cellulose and overlap the Si–O–Si bands.

**Fig. 15:**
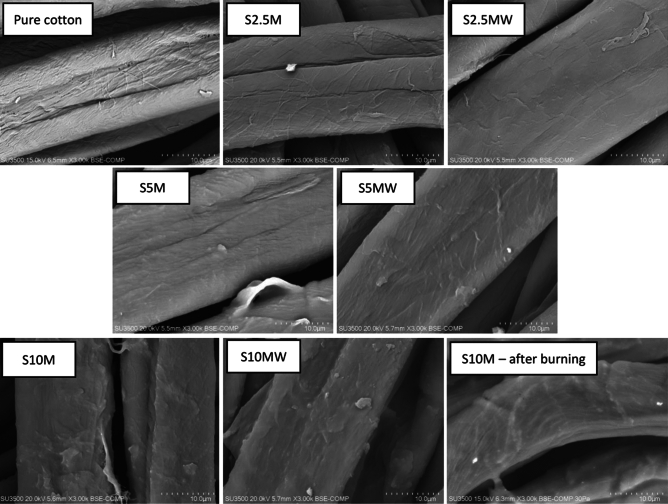
SEM images of pure cotton, modified cotton fabrics and fabric S10M after burning.

The cotton samples, including the sample of unmodified cotton as a reference sample, and sample S10M after burning were subjected to SEM microscopic analysis (Fig. 15).

In the first photo of pure cotton the varied fiber morphology typical of raw fabric is visible. In the subsequent photos clear differences resulting from the modifications can be seen. The photos of the modified samples show a layer of silane on the surface of the fibers. The photos show a homogeneous siloxane layer clearly surrounding the fibers. Analyzing the SEM photos, it can be assumed that the silane not only formed bonds with the fibers but also condensed between them, producing a siloxane layer. Moreover, it can be seen that as the concentration of silane in the modifying composition increases, the more distinct layer formed on the fiber surface. When using a 10% solution, single agglomerates also appear. It is important to note that the silane layer is clearly visible in the photos of washed samples and the photos look similar to unwashed samples. This proves the durability of the modifications after repeated washing. The last photo shows the S10M sample after burning. We can observe preserved cotton fibers. Modification promoted the formation of a compact char layer which can prevent further combustion by cutting off the heat and oxygen. SEM photos of the remaining samples are included in the Supplementary Information.

To confirm the presence of silane on the fiber surface, SEM–EDS elemental analysis was performed (Table 5).

**Table 5:** SEM–EDS results of modified samples before and after washing.

Sample	SEM–EDS									
	Before washing					After washing				
	C	O	Si	P	S	C	O	Si	P	S
Pure cotton	34.2	65.5	–	–	–	34.2	65.5	–	–	–
S2.5	35.6	61.3	0.7	0.8	1.5	36.1	60.5	0.8	0.7	1.6
S2.5M	36.1	60.1	0.8	0.9	1.9	34.9	61.5	1.0	0.8	1.7
S5	34.4	59.2	1.5	1.4	3.1	35.3	58.9	1.5	1.3	2.9
S5M	34.5	59.1	1.5	1.5	3.3	35.5	58.2	1.6	1.5	3.1
S10	34.6	54.0	2.7	2.7	5.6	34.8	54.3	2.5	2.6	5.5
S10M	33.9	54.4	2.6	2.9	6.0	33.4	55.1	2.7	2.8	5.8
S10M-after burning	45.2	34.4	6.9	8.3	3.5					

The results presented in Table 5 clearly confirm that the modification was successful. There are clearly visible differences in the elemental composition of modified fabrics compared to unmodified fabrics. Impregnated samples are characterized by the presence of silicon, phosphorus and sulfur in their composition. These are elements characteristic of silane used to reduce the flammability of fabrics. Therefore, the presence of the mentioned elements in the modified fabrics clearly confirms that the modification has occurred. The content of elements characteristic of the silane used increases with its percentage concentration in the modifying compositions. Moreover, mercerized samples have slightly higher contents of elements characteristic of the modifier compared to non-mercerized ones. These results correlate perfectly with previous analyses. Moreover, what is most noteworthy is that the elemental composition of unwashed and washed samples remains almost unchanged. The SEM–EDS analysis once again confirmed the durability of the modifications made. The process of washing 10 times did not cause any significant changes in the elemental composition of the samples. All element values of washed and analogous unwashed samples remain at a very similar level. A slight decrease in the content of elements after washing may be caused by the washing out of particles that did not form bonds with the fiber surface and only accumulated them. However, washing away this small amount of modifier did not result in any deterioration of the remaining parameters as a result of washing. The element ratio of cotton before and after combustion changed significantly. The weight percentage of C increases after burning from 33.9 to 45.2% indicating that the fibers were carbonized during combustion. The weight percentage of P and Si increases considerably after burning. Based on the obtained results, conclusions can be drawn regarding the mechanism of flame retardancy of the applied modification, that P and Si remain in condensed phase and promoted the formation of a char layer. Silicon-containing part form siliceous barrier and protect from heat flux and volatile formation^37^. The weight percentage of S decreases which may be related to the gas-phase flame retardant mechanism of sulfur.

The bending length (stiffness) and flexural rigidity of the pure cotton and the modified cotton samples are shown in Table 6.

**Table 6:** Bending length and flexural rigidity of pure cotton and modified cotton fabrics.

Sample	Bending length (mm)	Flexural rigidity (µJ/m)
Pure cotton	13.5	5.1
S2.5	14.0	5.7
S2.5M	14.0	5.7
S5	15.5	7.7
S5M	15.5	7.7
S10	15.5	7.7
S10M	15.5	7.7

From the results given in Table 6, one can realize that the applied modifications do not, to a great extent, influence the flexibility of the fabric. The fabric bending length was slightly increased with the increase of silane concentration and the process of mercerization has not influenced on additional increase in fabrics stiffness. The bending length for the mercerized fabric modified with a 10% solution (S10M) increases by only 2 mm as compared with the raw fabric. Further, the flexural rigidity of the modified fabrics is very near to that of the unmodified fabric. These results would indicate that the process of mercerization has not influenced the stiffness of the fabrics and modification using both 2.5%, 5% and 10% silane solutions does not significantly affect the stiffness of fabrics. The elasticity of the chemically modified fabrics is very important with regard to their usability.

## Conclusions

This work focused on the synthesis and modification of cotton fabric by phosphorus, sulfur and silicon-containing flame retardant. Silane containing triethoxysilyl and phosphate groups was designed and synthesized by hydrothiolation reaction. A compound with 90% isolation yield was obtained and characterized by NMR and FT-IR analyses. Cotton samples were modified using a simple, one-step sol–gel process. According to the pyrolysis-combustion flow calorimetry (PCFC) technique, the use of the obtained silane for cotton treatment can reduce heat release rate (HRR) from over 40% (2.5 wt.%) to almost 75% (10 wt.% of silane) compared to the raw fabric. After washing cycles, the created coatings demonstrated high resistance to washing. This spectacular effect proved that the alkoxy groups present in the structure of the silane modifier enabled the formation of covalent bonds on the fiber surface. The limited oxygen index values (LOI) increase with the concentration of silane in the modifying composition and ranged from 20 to 24.8% (20 wt.% of silane), compared to 18% for raw cotton. The TGA and DTG analyses indicate that the modified cotton fabrics exhibit significantly improved thermal stability compared to pure cotton, with higher residue values and lower degradation rates. Additionally, washing has minimal impact on the thermal properties of the modified fabrics, confirming the durability of the modification. FT-IR analyses confirmed the modification of the cotton surface. SEM images indicate that impregnated cotton samples were covered with a layer of silanes and that the layer was more distinct with increasing the concentration of silane in the modifying composition. Importantly, the modifier layer is also visible on washed samples and the photos look similar to unwashed ones. The SEM–EDS analysis once again confirmed successful modification of cotton fabrics. The impregnation process did not significantly stiffen or change the color of the cotton fabrics.

This study demonstrates that the novel silane compound synthesized via a simple one-step process effectively enhances the flame retardant properties of cotton fabrics.

### Supplementary Information

Supplementary Information.

## Data Availability

The datasets generated during the current study are available from the corresponding author on reasonable request.
