# Supplementary material for: Synthesis of phosphorus, sulfur and silicon-containing flame retardant via thiol-ene click reaction and its use for durable finishing of cotton fabric
Source: Sci Rep. 2024 Aug 29;14:20132. doi: 10.1038/s41598-024-71071-5 (PMC11362145; doi:10.1038/s41598-024-71071-5)
Supplement: Supplementary file 1 — Supplementary Information. [file 41598_2024_71071_MOESM1_ESM.pdf]

SUPPLEMENTARY INFORMATION

Synthesis of phosphorus, sulphur and silicon-containing flame retardant via thiol-ene click reaction and its use for durable finishing of cotton fabric

Anna Szymańska[1,*], Marcin Przybylak[1], Michał Dutkiewicz[1], and Hieronim Maciejewski[1,2]

[1]Poznań Science and Technology Park, Adam Mickiewicz University Foundation, Rubież 46, 61-612 Poznań, Poland

[2]Faculty of Chemistry, Adam Mickiewicz University, Uniwersytetu Poznańskiego 8, 61-614 Poznań, Poland

[*]anna.szymanska@ppnt.poznan.pl

Table of contents

1. NMR spectra 2
2. FT-IR spectrum 4
3. SEM images 5
4. Images of samples after LOI tests 7

1. NMR spectra

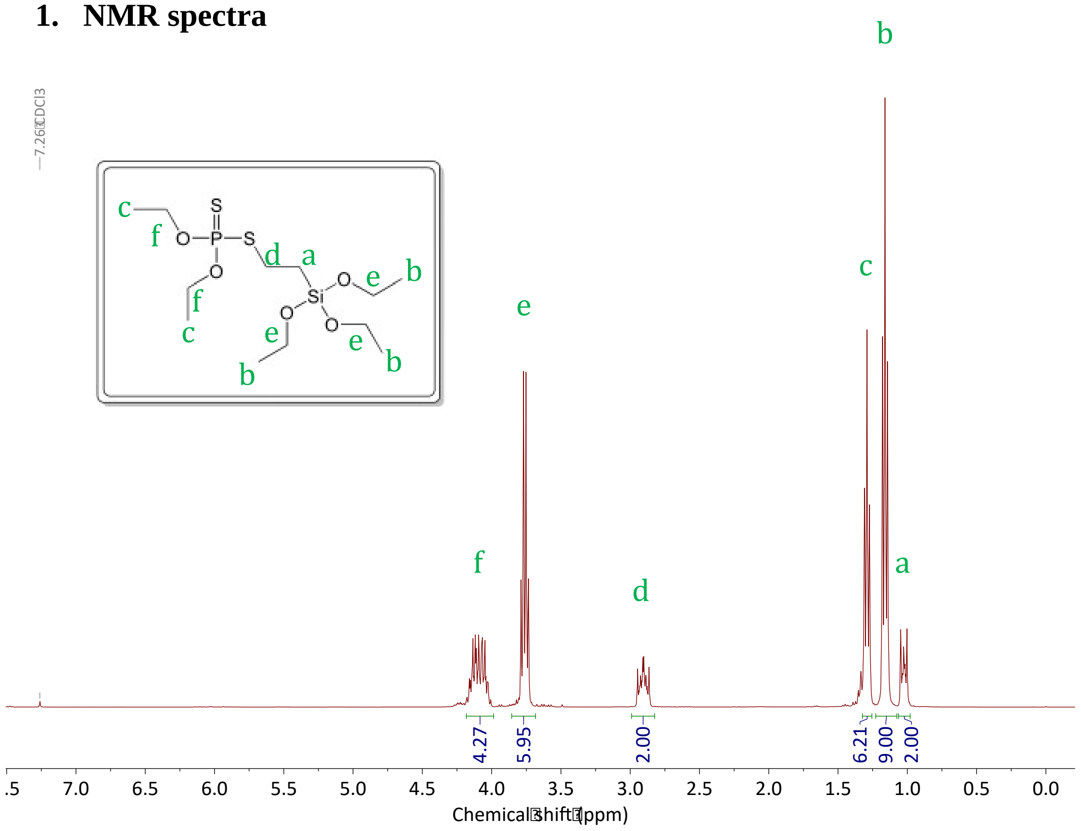

Figure S1. ^{1}H NMR spectra of *O,O'*-diethyl-S-[(2-triethoxysilyl)ethyl] phosphorodithioate.

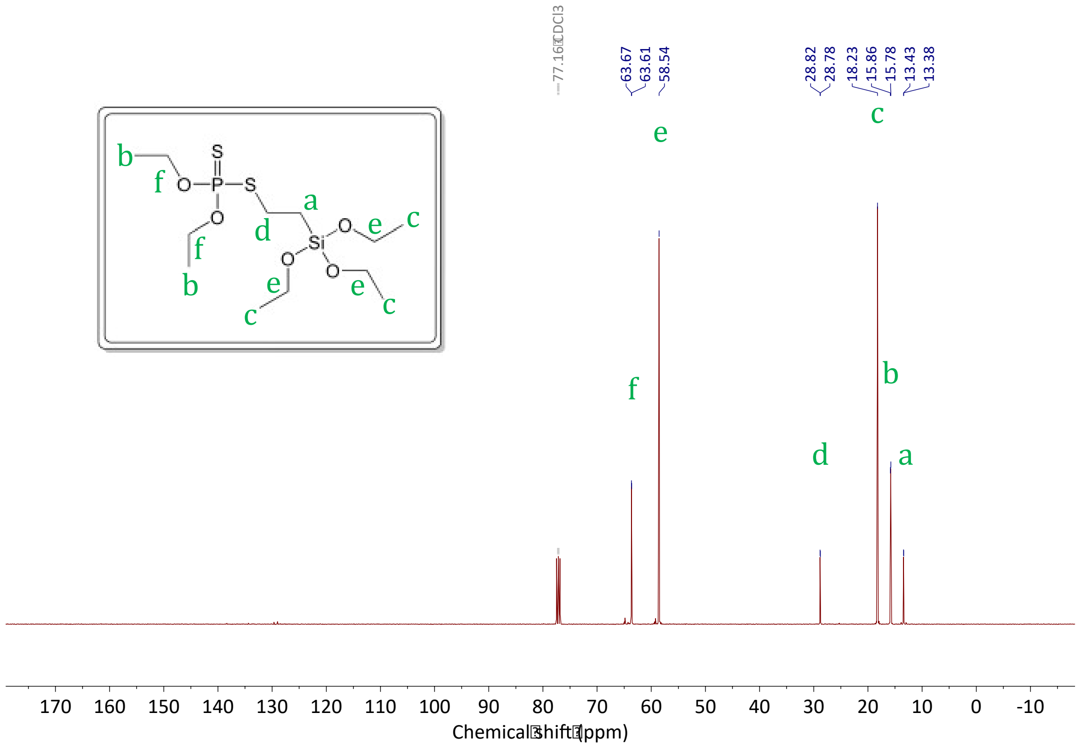

Figure S2. ^{13}C NMR spectra of *O,O'*-diethyl-S-[(2-triethoxysilyl)ethyl] phosphorodithioate.

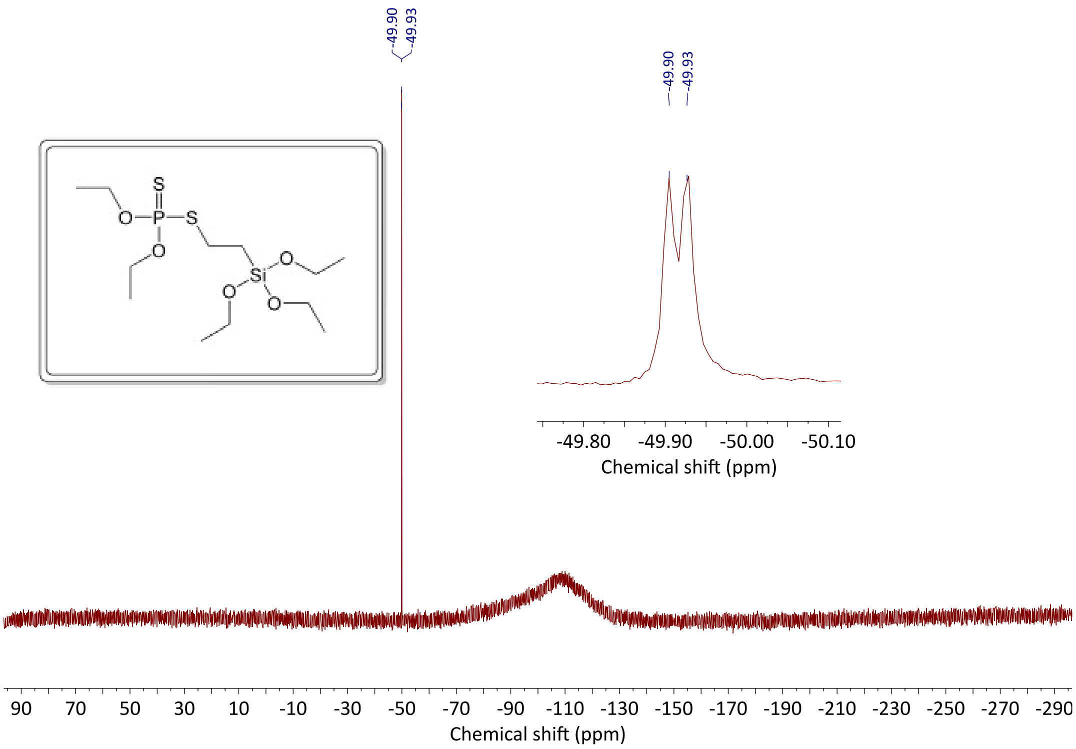

Figure S3. ^{29}Si NMR spectra of *O,O'*-diethyl-S-[(2-triethoxysilyl)ethyl] phosphorodithioate.

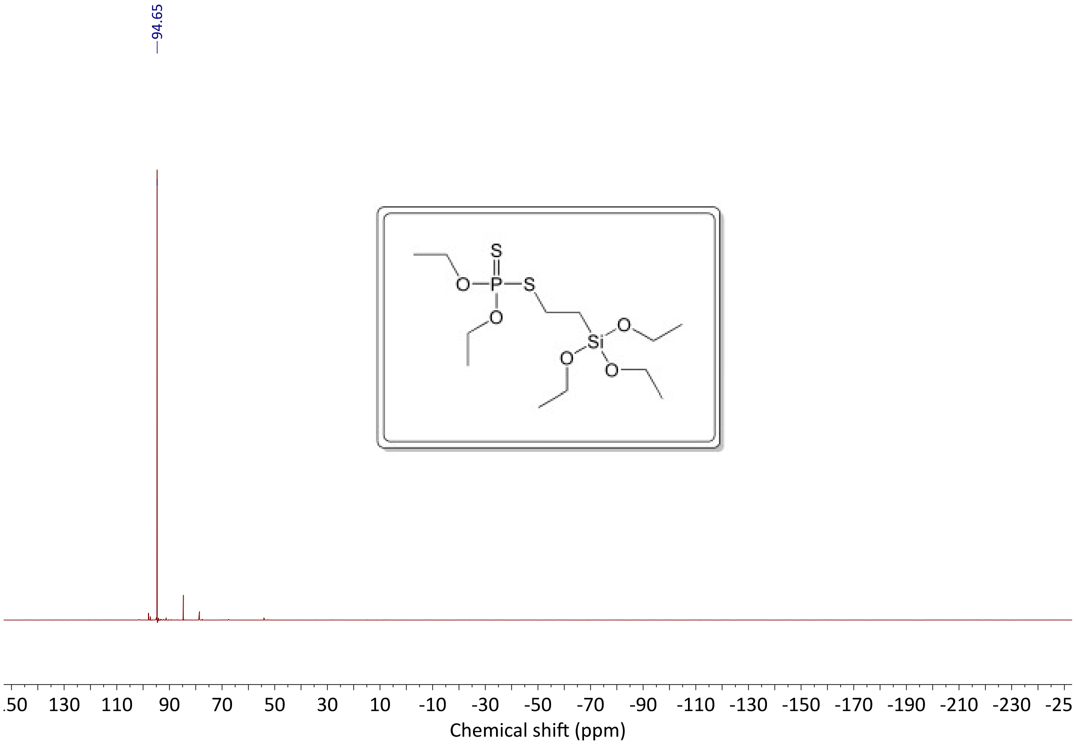

Figure S4. ^{31}P NMR spectra of *O,O'*-diethyl-S-[(2-triethoxysilyl)ethyl] phosphorodithioate.

2. FT-IR spectrum

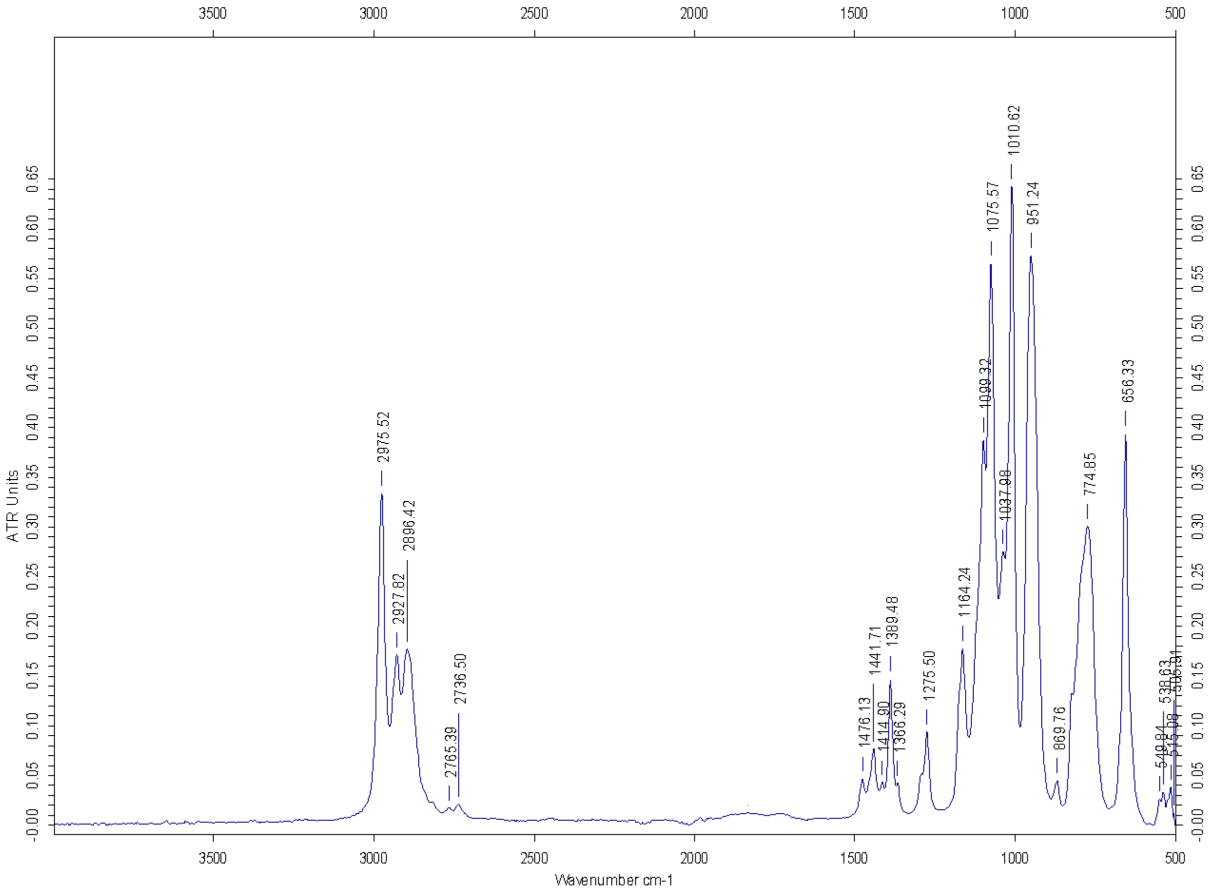

Figure S5. FT-IR spectrum of *O,O'*-diethyl-S-[(2-triethoxysilyl)ethyl] phosphorodithioate.

3. SEM images

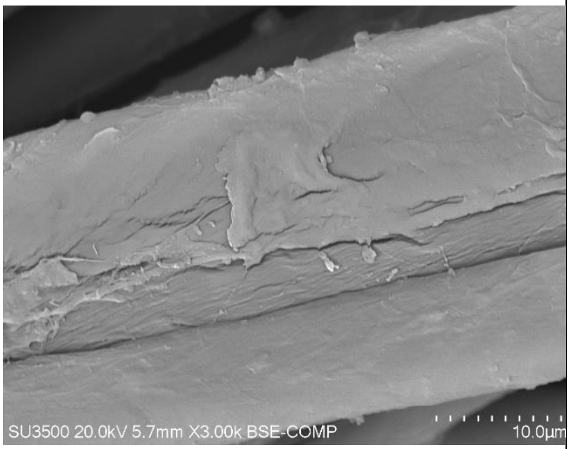

S2.5

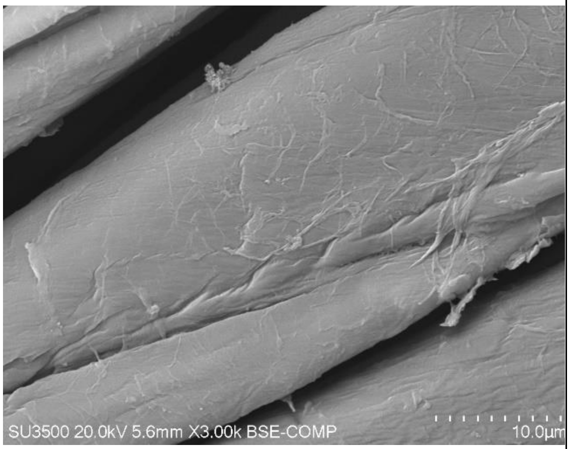

S2.5W

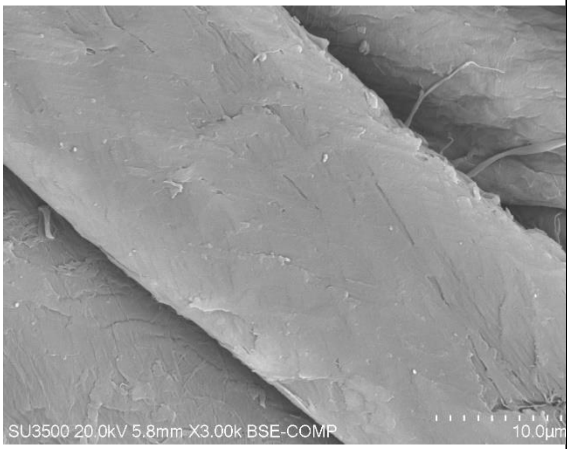

S5

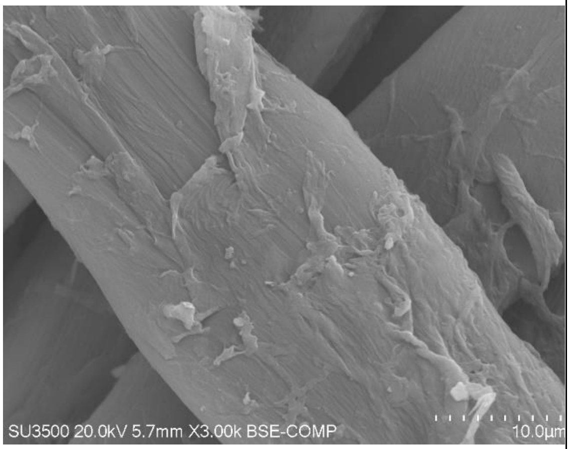

S5W

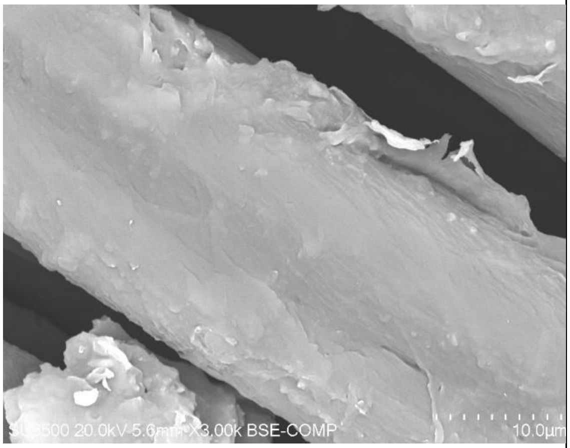

S10

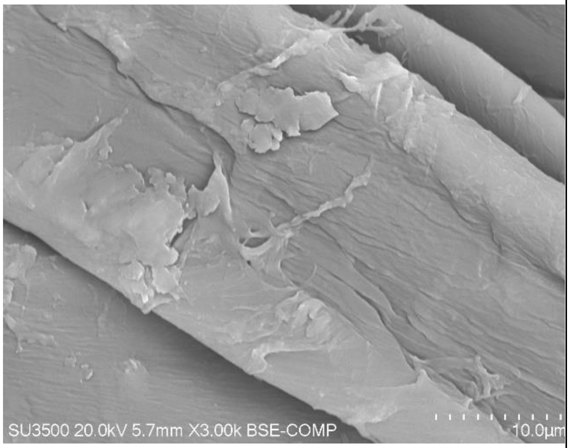

S10W

4. Images of samples after LOI tests

The photographs show samples after LOI analysis. The images were taken for samples with the highest LOI value for each type of sample. The LOI results are summarized in Figure 8. (10 – 10x washing cycles, 20 - 20x washing cycles, 30 - 30x washing cycles).

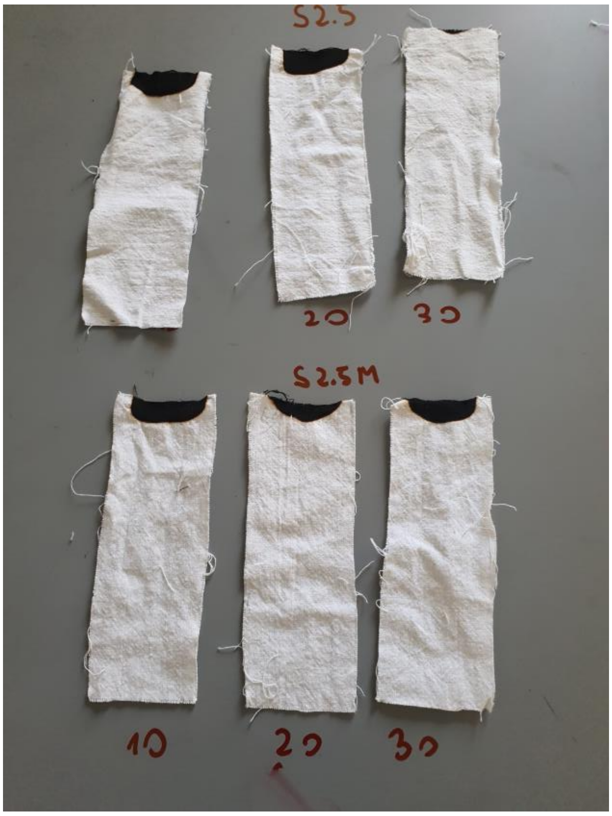

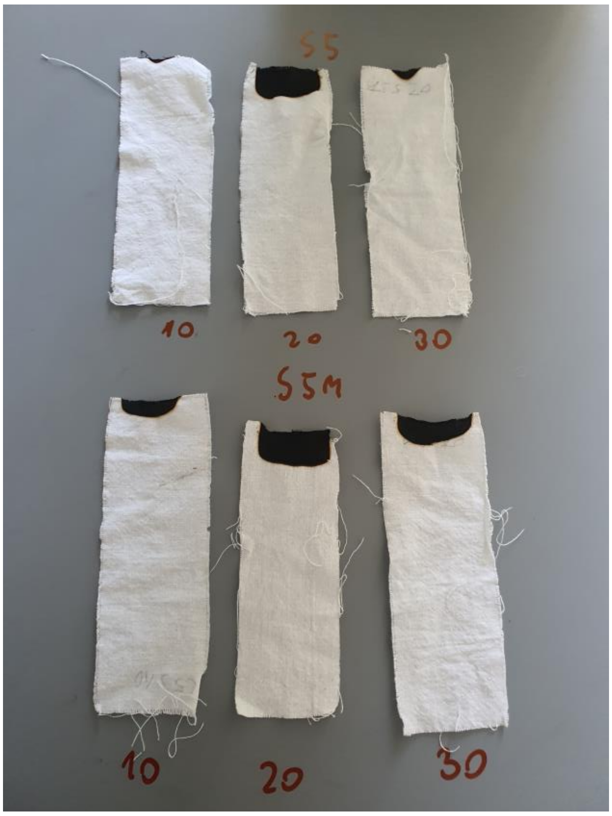
S5
10
20
30
S5M
10
20
30

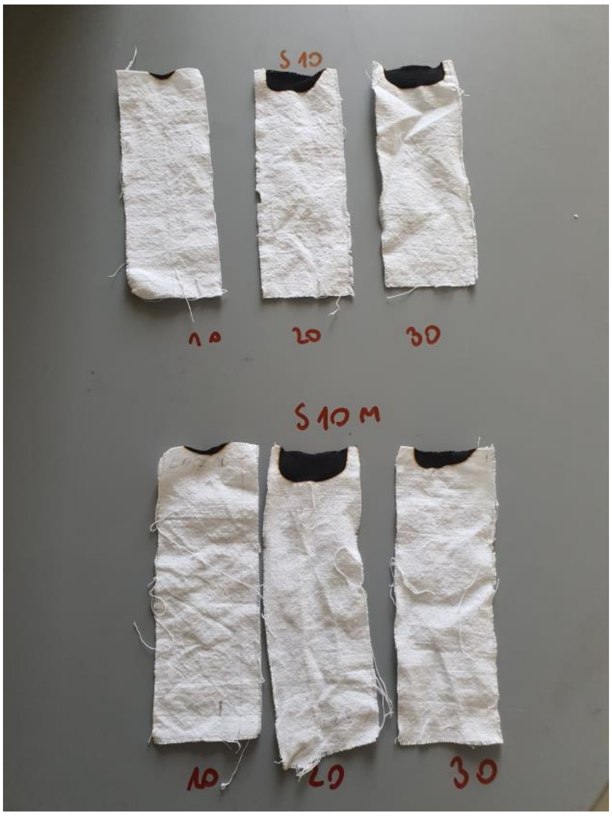
S10
20
30
S10M
10
20
30

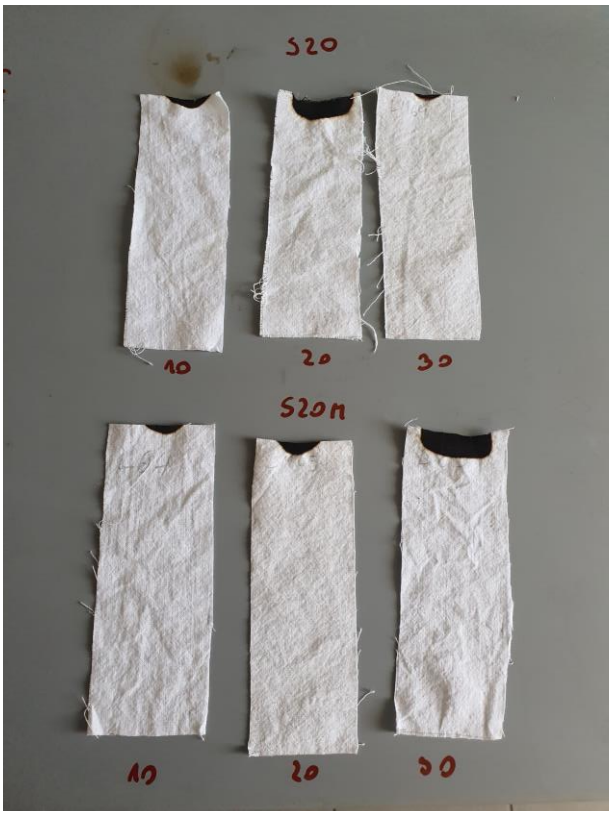
S20
10
20
30
S20M
10
20
30
